# Relationships between intracranial arterial dolichoectasia and small vessel disease in patients with ischaemic stroke: a systematic review and meta-analysis

**DOI:** 10.1007/s00415-023-12094-2

**Published:** 2023-11-29

**Authors:** Kitti Thiankhaw, Hatice Ozkan, Gareth Ambler, David J. Werring

**Affiliations:** 1 Division of Neurology, Department of Internal Medicine, Faculty of Medicine, Chiang Mai University, Chiang Mai, Thailand; 2 Stroke Research Centre, Department of Brain Repair and Rehabilitation, University College London Queen Square Institute of Neurology, London, UK; 3 Department of Statistical Science, Faculty of Mathematical & Physical Sciences, University College London, London, UK

**Keywords:** Ischaemic stroke, Dolichoectasia, Small vessel disease, Cerebral microbleeds, White matter hyperintensities

## Abstract

**Background:**

Intracranial arterial dolichoectasia (IADE) is a common arterial finding of dilation, elongation, or both, affecting large intracranial vessels, and associated with vascular risk factors, including hypertension. Associations of IADE with neuroimaging cerebral small vessel disease (CSVD) may be relevant for diagnosis and prognosis in patients with stroke. The study aimed to conduct an updated systematic review and meta-analysis of observational studies to investigate the relationships of IADE with well-defined CSVD markers in patients with ischaemic stroke.

**Methods:**

We systematically searched PubMed, Embase, and Scopus for studies on IADE in ischaemic stroke patients with fulfilling predefined inclusion criteria. We pooled data to conduct a meta-analysis to compare the prevalence of SVD markers between patients with and without IADE groups using risk ratios (RRs) and 95% confidence intervals (CIs).

**Results:**

From 157 retrieved abstracts, we included six studies from seven publications comprising 6102 patients with ischaemic stroke. The mean age of patients was 52.8 years, and 3691 (60.5%) were male. IADE was diagnosed in 11.4% (95% CI 8.9–13.9) (761) of included patients; 51.8% (3160) had hypertension. Compared to patients without IADE, individuals diagnosed with IADE had a significantly increased prevalence of lacune (RR 1.67, 95% CI 1.36–2.06, *P* < 0.01, *I*^*2*^ = 0.00%), cerebral microbleeds (CMBs) (RR 2.56, 95% CI 1.53–4.28, *P* < 0.01, *I*^*2*^ = 84.95%) and white matter hyperintensities (WMHs) (RR 2.17, 95% CI 1.84–2.56, *P* < 0.01, *I*^*2*^ = 0.00%).

**Conclusions:**

In patients with ischaemic stroke, IADE is associated with a higher prevalence of CSVD markers, including lacunes, CMBs, and WMHs. Further studies are needed to clarify the mechanisms underlying these associations and their potential relevance for the understanding, diagnosis, and treatment of CSVD.

**Supplementary Information:**

The online version contains supplementary material available at 10.1007/s00415-023-12094-2.

## Introduction

Intracranial arterial dolichoectasia (IADE) is a common arteriopathy affecting large intracranial vessels [1]. It is characterised by abnormal fusiform dilatation (ectasia) and elongation or tortuosity (dolichosis) of the intracranial arteries, mainly involving basilar arteries (BA) in approximately 80% of all cases [2, 3]. Because of arterial elongation and enlargement, patients with IADE can present with various clinical syndromes, including cerebral ischaemic symptoms due to thromboembolism or local compression (e.g., affecting the cranial nerves or brainstem); obstructive hydrocephalus; or subarachnoid haemorrhage (SAH) [4–6]. Its prevalence in the general population is relatively low, ranging from 0.06% to 5.8%, but its prevalence has been reported to range from 3 to 18% in patients with ischaemic stroke [1, 7] [8–11].

Recent studies have demonstrated a relationship between cerebral small vessel disease (CSVD) and IADE, which might be important to better understand disease pathogenesis and clinical relevance for diagnosis, prognosis, and treatment in people with cerebrovascular disease [3, 12]. Advancing age, hypertension, and male sex are associated with IADE, but it is not certain whether these fully explain the observed associations [13, 14]. Studies on patients with lacunar stroke revealed that IADE was significantly more common in ischaemic stroke attributed to small vessel occlusion than to athero-thromboembolism (36% vs. 19%) and patients with (compared to those without) severe white matter disease (34% vs. 19%) [7, 15]. However, previous studies are limited in providing a definitive estimate of the strength and consistency of any association with IADE with CSVD. We, therefore, did an updated systematic review and meta-analysis of observational studies to investigate the relationships of IADE among magnetic resonance imaging (MRI)-defined CSVD markers in patients with ischaemic stroke.

## Methods

### Protocol and registration

We conducted this systematic review according to the Cochrane Handbook for Systematic Reviews of Interventions, the Preferred Reporting Items for Systematic Reviews and Meta-Analyses (PRISMA) 2020 statement, and the PRISMA extension statement for reporting of systematic reviews that incorporate network meta-analyses of healthcare interventions (Supplementary material: PRISMA 2020 checklist) [16–18]. The protocol of this systematic review was prospectively registered at PROSPERO (registration ID: CRD42023417010).

### Search strategies and eligible criteria

We systematically searched PubMed, Embase, and Scopus from inception until March 23, 2023. The search strategies were derived from the keywords ‘dolichoectasia’, ‘stroke’, and ‘small vessel disease’ (Appendix Table S1). The searches and the study selection had no limitation on language, publication year, or publication status. The inclusion criteria for the included study were as follows: (i) participants—patients with ischaemic stroke; (ii) exposure—participants with IADE; (iii) comparator—participants without IADE; (iv) outcomes—reporting the outcomes of small vessel disease (SVD) markers, including lacune, cerebral microbleeds (CMBs), leukoaraiosis or white matter hyperintensities (WMHs), and perivascular spaces (PVSs).

### Study selection and data extraction

The titles and abstracts were independently screened, and the full-text articles of the retrieved records were assessed to select eligible studies. Any discrepancies between their results were resolved through a consensus discussion between two reviewers (K.T. and H.O.). The primary outcome of the study was the prevalence of SVD markers among ischaemic stroke patients with IADE compared to those without IADE. Data were extracted from the included studies by reviewers into a standardised spreadsheet form. K.T. extracted the study information and data; H.O. rechecked the extracted data to ensure its accuracy. The extracted data included study ID (first author and year of publication), types of stroke, number of patients, age, gender, comorbidities, number of participants who presence of IADE and SVD markers (lacune, CMBs, WMHs, état criblé (EC), and PVSs). We extracted the most complete and updated data for studies with multiple publications. Continuous data reported as the median and interquartile range (IQR) were converted to mean and standard deviation (SD) using a method proposed by a previous study [19].

### Quality of studies and risk of bias assessment

The quality of each included study was independently assessed by K.T. and H.O. using the Newcastle–Ottawa Quality Assessment Scale (NOS) for case–control and cohort studies [20]. Any discrepancies (or any disagreements) between their assessments were resolved through a consensus discussion with the senior author (D.J.W.). The weighted kappa statistic was calculated to measure interrater agreement and revealed kappa values of 0.65 (standard error (SE) 0.25), which can be interpreted as a substantial agreement between the authors (0.61–0.80) [21]. The quality domains being assessed included selection, comparability, and outcome. The NOS stars thresholds were converted to Agency for Health Research and Quality (AHRQ) standards. Good quality was characterised by 3 or 4 stars in the selection domain and 1 or 2 stars in the comparability domain, and 2 or 3 stars in the outcome domain. The median of the numbers of stars obtained from included studies was calculated for each pooled result. A median of 4–5 stars or 0–3 stars was considered a high or very high risk of bias, respectively (more stars indicated a low risk of bias). Funnel plots were used to explore the presence of small-study effects often associated with publication bias. The symmetrical plot represented the absence of small study effects.

### Data synthesis and statistical analysis

We conducted a meta-analysis to compare the prevalence of individual SVD markers between patients with and without IADE groups using risk ratios (RRs) and 95% confidence interval (CI). We estimated the pooled individual prevalence utilising random-effects meta-analysis with a restricted maximum likelihood (REML) method [22]. A RR > 2 or < 0.5 and > 5 or < 0.2 were considered large and very large effect sizes, respectively [23]. Cochrane Chi-square (*Q* test) and *I*^*2*^ statistics were utilised to test the heterogeneity of each dataset, and *I*^*2*^ statistic of 51%–75% or greater than 75% was considered moderate and high heterogeneity, respectively [24]. Where we identified substantial heterogeneity, we performed meta-regression analyses and produced bubble plots to examine the possible study-level covariates of mean age, the quality of the study, the percentage of male participants, SVD markers and hypertension. Publication bias was investigated using the funnel plot, and the Egger regression-based test was applied to test for funnel-plot asymmetry. A *P* value less than 0.05 was considered statistically significant. All statistical analyses were performed using licensed Stata statistical software version 16.1 (Stata Statistical Software: release 16.1, Stata Corporation, College Station, TX, 2019).

### Quality assessment of cumulative evidence

The certainty of evidence was independently rated using the Grading of Recommendations Assessment, Development and Evaluation (GRADE) guidelines [25]. The quality of a body of evidence was initially graded as low because of the observational study designs, and then downgraded or upgraded based on the risk of bias, publication bias, imprecision (random error), inconsistency, indirectness, large effect, dose response, and effect of plausible residual confounding [26]. This quality reflected the confidence in the effect-size estimate (RR), and overall certainty for each outcome was classified as very low, low, moderate, or high.

## Results

### Study selection, characteristics, and risk of bias within studies

**Fig. 1:**
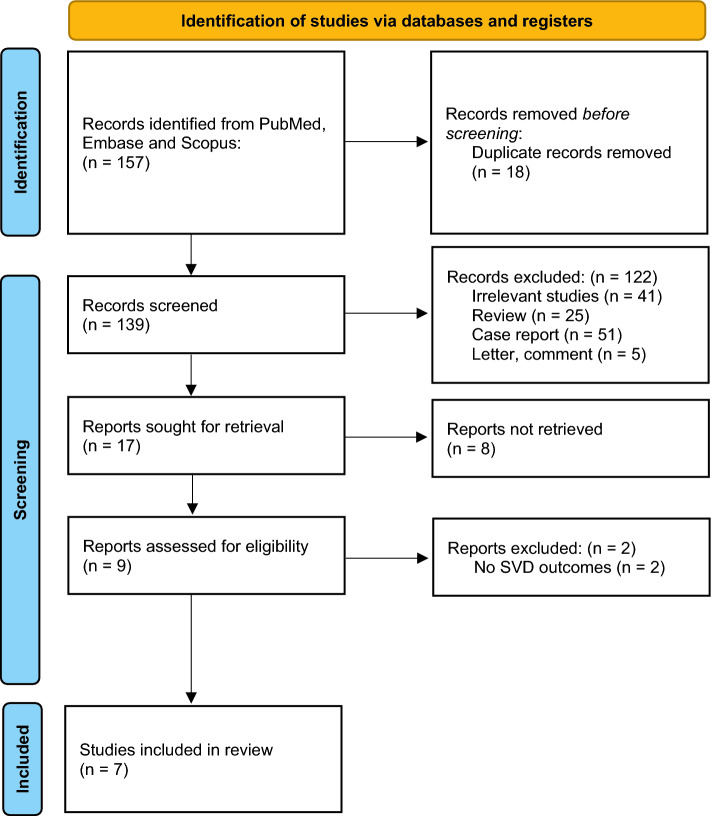
PRISMA 2020 flow diagram summarising the study selection process from PubMed, Embase, and Scopus searches for the studies included in the meta-analysis. *SVD* small vessel disease

We retrieved a total of 157 abstracts. After removing duplicated records using an automation tool (The Systematic Review Accelerator: SRA) [27], abstract screening, and assessing 17 full texts, we included six case–control and observational cohort studies from seven publications comprising 6102 patients with ischaemic stroke (Fig. 1) [7, 12, 13, 15, 28–31]. Most of the included studies used computed tomography angiography (CTA) or magnetic resonance angiography (MRA) as imaging modalities for diagnosing IADE, however, in a study by Pico et al. brain-autopsy was used, while the study of Brutto et al. used a semiautomatic vessel calculation method, which IADE was performed using automated software, and used to calculate the vessel diameter, length and tortuosity index (TI) [12, 28]. The Smoker criteria were most commonly used to define vertebrobasilar dolichoectasia (VBD), except in a study by Thijs et al. that utilised another definition (Table 1) [13].

**Table 1:** Study characteristics and outcomes of SVD markers in ischaemic stroke patients with IADE (ordered by year of publication)

Study	Stroke types	No. of patients	Age (y), mean (SD)	Male, no. (%)	HT, no. (%)	+ IADE, no. (%)	‒IADE, no. (%)	SVD markers in + IADE group, no. (%)						Imaging modality	IADE definition
								Lacune	Multilacunes^†^	WMHs^‡^	EC	CMBs	SVD		
Pico 2003 [7]	IS	510	60.0 ± 13.2	318 (62.4)	330 (64.7)	63 (12.4)	447 (87.6)	23/63 (36.5)*	NA	NA	NA	NA	NA	MRI and CTA	Smoker criteria^§^
Pico 2005 [15]	IS	510	60.0 ± 13.2	318 (62.4)	330 (64.7)	63 (12.4)	447 (87.6)	NA	32/63 (50.8)*	20/59 (33.9)*	19/60 (31.7)*	NA	NA	MRI and CTA	Smoker criteria
Pico 2007 [28]	Stroke	381	72.4 ± 12.0	207 (54.3)	214/375 (57.1)	23 (6.0)	358 (94.0)	11/23 (47.8)*	8/23 (34.8)*	NA	5/22 (22.7)	NA	10/22 (45.5)*	Brain-autopsy study	
Park 2013 [29]	IS	182	65.5 ± 11.6	96 (52.7)	125 (68.7)	24 (13.2)	158 (86.8)	NA	NA	NA	NA	16/24 (66.7)*	NA	MRI and MRA	Smoker & Ubogu criteria^∥^
Thijs 2017 [13]	IS	3850	46.0 ± 8.0	2278 (59.2)	1773 (46.1)	508 (13.2)	3342 (86.8)	NA (23.0)	NA	97/508 (19.0)*,^¶^	NA	33/202 (16.3)*	NA (18.1)*	MRI	**
Yin 2021 [30]	IS (ICAS)	469	60.2 ± 11.3	339 (72.3)	297 (63.3)	61 (13.0)	408 (87.0)	NA	19/61 (31.1)*	26/61 (42.6)*	NA	NA	NA	MRI and MRA	Smoker criteria
Osama 2022 [31]	IS	200	65.2 ± 12.9	135 (67.5)	91 (45.5)	19 (9.5)	181 (90.5)	NA	NA	NA	NA	16/19 (84.2)*	NA	MRI and MRA	Smoker criteria
Overall		6102	52.8 ± 13.2	3691 (60.5)	3160 (51.8)	761 (11.4)	5341 (88.6)	34/86 (39.5)	59/147 (40.1)	143/628 (22.8)	24/82 (29.3)	65/245 (26.5)	10/22 (45.5)		

*EC* État criblé; *CMBs* cerebral microbleeds; *CTA* computed tomography angiography; *HT* hypertension; *IADE* intracranial arterial dolichoectasia; *ICAS* intracranial atherosclerosis; *IS* ischaemic stroke; *MRA* magnetic resonance angiography; *MRI* magnetic resonance imaging; *NA* not applicable; *SD* standard deviation; *SVD* small vessel disease; *WMHs* white matter hyperintensities

*Statistical significance compared to patients without IADE (*P* < 0.05 or < 0.001)

**The tortuosity of the BA was rated as none, mild (some tortuosity of BA with a deviation from the midline of > 5 mm to ≤ 10 mm), moderate (deviation of BA from midline by > 10 mm and diameter > 5 mm), or severe (tortuosity with an impression of brain stem and diameter > 10 mm). The maximum BA artery diameter was also directly measured at its maximum on axial MRI scans

^†^Multilacunar was defined as the number of lacunar infarctions greater than one

^‡^At least grade 2 or 3 WMHs in the deep or periventricular white matter

^§^Vertebrobasilar dolichoectasia (VBD) was defined as both ectasia and dolichosis, which were simultaneously observed in each patient. Ectasia was defined when the diameter of the basilar artery (BA) was > 4.5 mm at any point along its course. Dolichosis of the basilar artery was considered when: it lay lateral to the margin of clivus or dorsum sella or was bifurcated above the plane of the suprasellar cistern

^∥^VA on intracranial MRA was considered elongated if the length was > 23.5 mm. Any portion of the VA with a deviation > 10 mm perpendicular to a straight line joining its intracranial entry point to the BA origin was considered abnormal

^¶^Combined deep and periventricular white matter

The mean age of patients was 52.8 years (SD 13.2), and 3691 (60.5%) were male. 3160 of 6096 (51.8%) ischaemic stroke patients had hypertension, and IADE was diagnosed in 11.4% (95% CI 8.9–13.9), 761 of included patients. The prevalence of IADE affecting the anterior circulation alone was 4.1%, which is lower than previous studies (13%) because some enrolled studies focused on VBD only. In terms of SVD markers, no studies reported all neuroimaging markers. Each SVD marker was reported by three studies, except two studies for EC, defined as dilatation of PVSs of the lenticulostriate or the white matter small arteries. Lacunes were the most common SVD neuroimaging marker among ischaemic stroke patients with IADE, being found in 34 in 86 patients (39.5%) (Table 1).

To assess the risk of bias, we deducted one point from the selection domain from all studies because the unexposed group was drawn from hospital controls within the same community as exposed, but derived from a hospitalised population; we also deducted one point from the comparability domain of one study in which the age or gender between groups was different or unknown. The overall NOS scores of all included studies ranged between 5 and 7 points (out of a maximum of 9 points) (Appendix Table S2). The included studies were rated fair quality according to the AHRQ standards, except those by Pico et al. [7, 15], which were graded as good quality.

### Risk of SVD markers among AIS patients with IADE

All included studies found that ischaemic stroke patients with IADE (compared to those without) had a higher prevalence of SVD markers (Table 1). From seven publications included in the systematic reviews, we removed a study by Pico et al. in 2003 before performing a meta-analysis because they used data from the GENIC (the Etude du Profil Genetique de l’Infarctus Cerebral) Study, the same cohort as described in their publication from 2005. We compared the risk of SVD markers between groups within the whole sample for lacunes, CMBs, and WMHs. No studies investigated PVS.

**Fig. 2:**
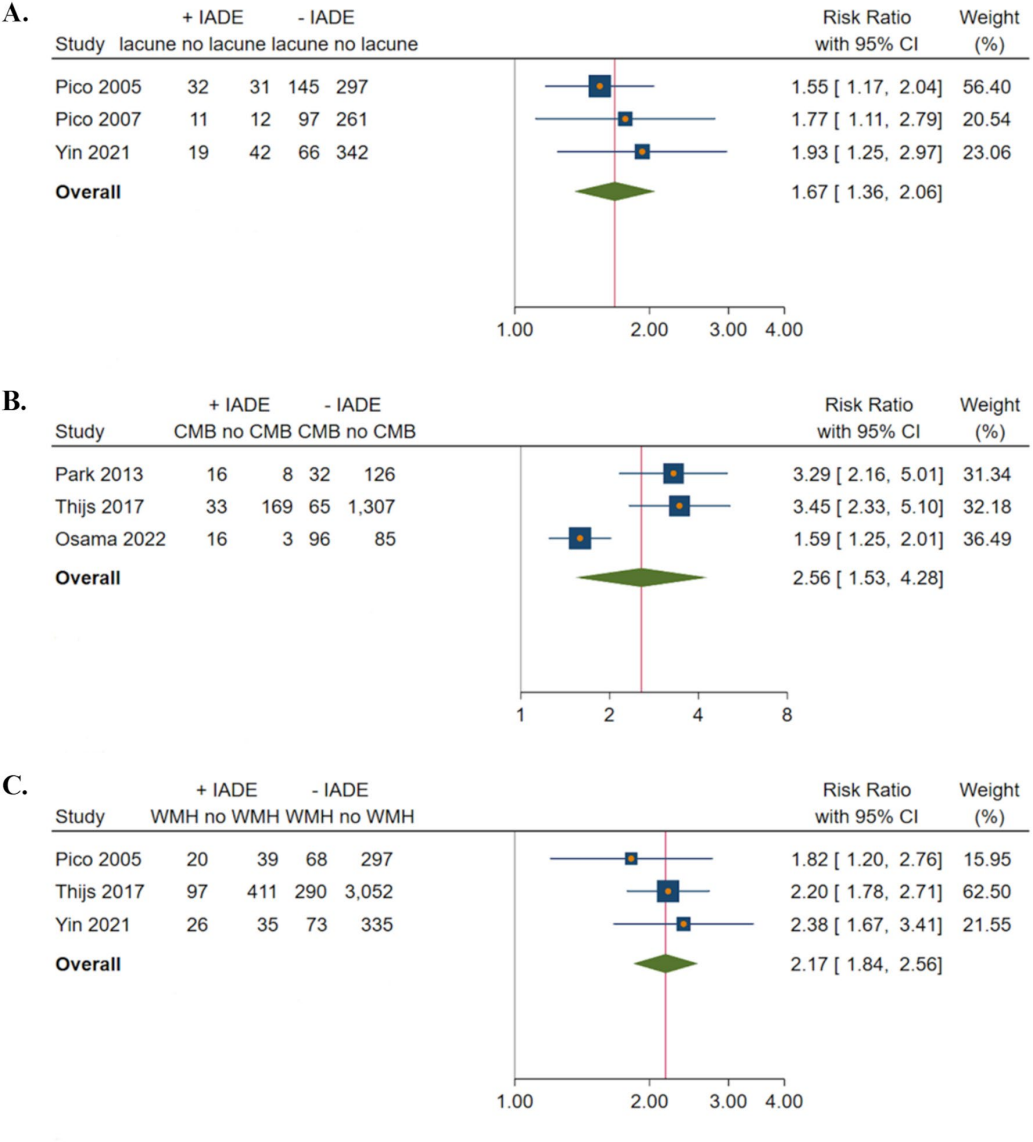
Forest plots of the risk (RR and 95% CI) of small vessel disease markers in ischaemic stroke patients with intracranial arterial dolichoectasia (**A**). lacunes (**B**). CMBs (**C**). WMHs. *CI* confidence interval; *CMBs* cerebral microbleeds; *IADE* intracranial arterial dolichoectasia; *WMHs* white matter hyperintensities

Compared to patients without IADE, individuals with IADE more often had lacunes (3 studies, RR 1.67, 95% CI 1.36–2.06, *P* < 0.01, *I*^*2*^ = 0.00%, *P* = 0.69 for the test for heterogeneity) (Fig. 2A), CMBs (3 studies, RR 2.56, 95% CI 1.53–4.28, *P* < 0.01, *I*^*2*^ = 84.95%, *P* < 0.01 for the test for heterogeneity) (Fig. 2B) and WMHs (3 studies, RR 2.17, 95% CI 1.84–2.56, *P* < 0.01, *I*^*2*^ = 0.00%, *P* = 0.62 for the test for heterogeneity) (Fig. 2C).

### Meta-regression analyses for handling heterogeneity and publication bias

**Fig. 3:**
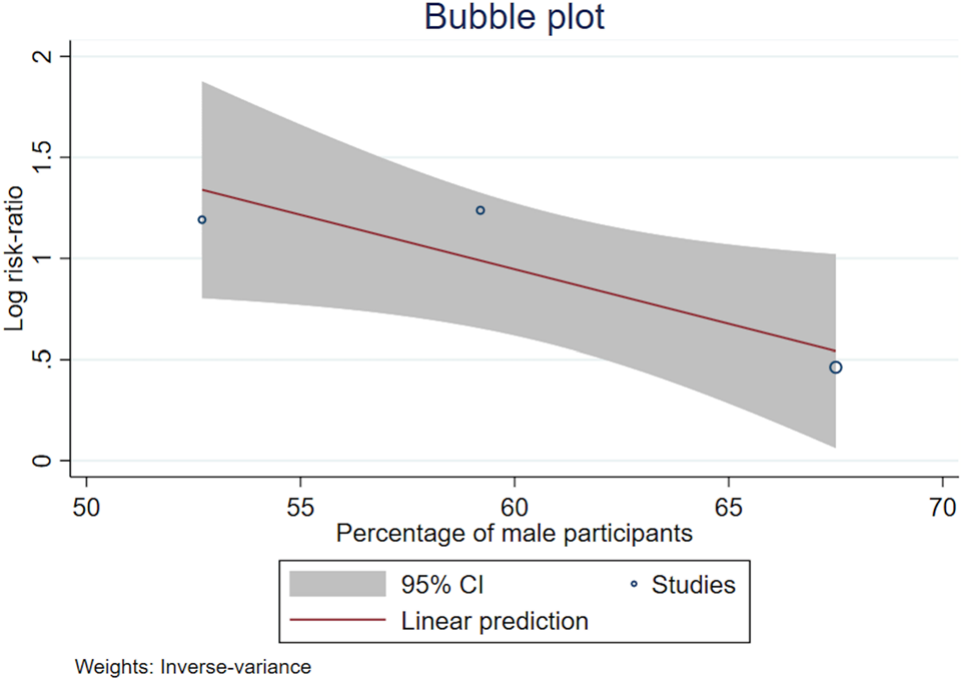
Bubble plots with fitted meta-regression lines of the risk of cerebral microbleeds and the percentage of male participants. The size of the bubbles represents the precision of the studies. *CI* confidence interval

Because of the significant heterogeneity of the prevalence of CMBs, we performed five meta-regression analyses to investigate whether this was associated with mean age, the quality of the study, the percentage of male participants, SVD markers, and hypertension. There was a statistically significant inverse relationship between the magnitudes of the effect sizes (CMBs) and the proportions of male participants: the greater the proportion of men, the smaller the risk of CMBs (coefficient − 0.05, SE 0.03, 95% CI − 0.11 to − 0.00, *P* = 0.045) (Fig. 3). After adjusting for the proportions of male participants, we determined that the residual heterogeneity between studies was 57.43%, down from 84.95%. There was no significant association between the risk of CMBs and the quality of enrolled studies, the mean age of participants, the percentage of CMBs, or hypertension (Appendix Table S3).

Appendix Fig. S1A–C shows the funnel plots for publication bias for each CSVD marker. The regression-based Egger test for small-study effects found that the funnel plot was asymmetric in CMBs outcome (a *z* statistic of 3.93 and a *P* < 0.001). This plot is based on only three observations, all suggesting a high prevalence of CMBs in IADE. No apparent asymmetry could be observed in funnel plots for lacunes and WMHs.

### Quality of cumulative evidence

The quality of evidence and its evaluation process are summarised in Appendix Table S4. The initial quality of evidence was considered low for all results because all included studies were observational. The results drawn from the CMBs outcome, which contained very high heterogeneity, were rated as a very high inconsistency (downgraded by -2 levels). The heterogeneity is being driven by the RRs. Although three articles on CMBs outcome focused on VBD, a study by Thijs et al. used data from stroke in young Fabry patients’ cohort, which resulted in differences among exposed and unexposed groups. No indirectness concern was found in any dataset (outcome). The significant results (imprecision) of all datasets (outcomes) resulted in no change in imprecision. Significant publication bias using Egger’s test further downgraded the certainty of evidence for the CMBs outcome by two levels. Large effect sizes were observed in CMBs and WMHs outcomes and led to an upgrade by one level. Taken together, the overall quality of evidence was rated from very low to moderate.

## Discussion

In our updated systematic review and meta-analysis, we found that 11.4% (95% CI 8.9–13.9) of ischaemic stroke patients had IADE, comparable to previously published data in ischaemic stroke populations [1–3, 6]. Patients with IADE were more likely than patients without IADE to have neuroimaging markers of CSVD, including lacunes, severe leukoaraiosis, CMBs, and WMHs. The included studies already adjusted for potential confounding factors, namely, age, sex, hypertension, ischaemic heart disease or previous myocardial infarction, leukoaraiosis, and stroke subtype, and the finding remained significant [7, 13, 15, 28–30]. Therefore, the associations of IADE with CSVD markers might be independent of these confounders or vascular risk factors.

Our results confirm an association between IADE and CSVD, consistent with the previous cohorts and case–control studies using clinical, imaging, and neuropathological evidence [7, 13–15, 28, 29, 32, 33]. Building on previous work, we show a consistent association of IADE with all of the CSVD markers we investigated and have been able to provide more precise estimates of the increased risk of CSVD associated with IADE. A key question is whether this association is simply due to shared vascular risk factors (e.g., hypertension, diabetes), or whether there are shared aspects of pathophysiology independent of these; our findings indicate that the latter explanation should be considered. Several underlying mechanisms have been proposed to explain the relationship between IADE and CVSD [34–36]. One hypothesis is that pathways associated with blood vessel structure and remodeling are relevant. For example, matrix metalloproteinase (MMP) and tissue inhibitors of metalloproteinase (TIMP) are associated with inflammation and remodeling of extracellular matrix and have been studied in relation to the linkage between CSVD and IADE [35, 37]. In 510 ischaemic stroke patients, MMP-3 was associated with IADE, suggesting a significance for MMPs in the formation of intracerebral dilative arteriopathy [37]. A study by DP Zhang et al. on 212 patients confirmed an apparent association and found elevated serum MMP-9 and the ratio of MMP-9 to TIMP-1 (MMP-9/TIMP-1) in vertiginous patients with VBD. Moreover, VBD patients with high-grade WMHs also had significantly high levels of TIMP-1, which correlated with the length and TI of the basilar artery [35]. MMP might also influence the risk of CSVD; one previous study suggests that mice models exposed to MMP-2 inhibitor exhibited lessened white matter lesions and microglia and astroglia activation following chronic cerebral hypoperfusion [38], while clinical studies in ischaemic stroke patients with a high burden of CSVD found an independent association with elevated MMP-9 and TIMP-4 [39, 40]. These results imply that MMP and TIMP might have a role in the pathogenesis of IADE (including VBD) and WMHs or other CVSD markers, with potential relevance for understanding, preventing, and treating cerebrovascular diseases.

IADE may be relevant clinically due to the potential risk of ischaemic stroke. A case–control study of patients with basilar dolichoectasia with and without ischaemic stroke found associations with reduced blood flow velocity, a multi-infarct pattern, and haemodynamic changes related to city and atheromatous alterations in the affected vessels have been proposed as mechanisms of ischaemic stroke (mainly occurring in the pons, but also the thalamus, midbrain and occipital lobes) in patients with BA dolichoectasia [34]. It can be implied that haemodynamic alterations may play an essential part in dolichoectasia [3].

Of the CSVD markers we investigated, cerebral microbleeds (CMBs) were the most strongly associated with IADE. CMBs are small (generally 2–10 mm), round or ovoid hypointensity lesions detected on T2*-gradient echo (GRE)/susceptibility-weighted imaging (SWI) and commonly found in patients with CSVD, including hypertensive arteriopathy (arteriolosclerosis) and cerebral amyloid angiopathy (CAA) [41, 42]. Our results confirm the previously reported associations between CMBs and large vessel dilatative arteriopathy, IADE and its subset (VBD), in various populations including ischaemic stroke [32, 33, 43]. The degree of basilar artery tortuosity, BA dolichosis, was independently associated with deep CMBs (Odds ratio (OR) 4.14, *P* = 0.002) [32]. In patients with VBD, CMBs are more frequently documented in the posterior circulation brain regions, including the cerebellum, thalamus, and occipital lobe [43] and a high CMBs burden (> 10 CMBs) was more frequent in vascular territories supplied by vessels arising from dolichoectatic vessels in the posterior region [31]. This anatomical link between large and small artery pathology is consistent with either shared pathophysiological mechanisms or vulnerability rather than simply shared risk factors [29, 31, 36, 43]. Importantly, results from meta-regression of gender heterogeneity on the influencing of CMBs in AIS patients with IADE showed that gender might not affect the incidence or burden of CMBs, in agreement with previous studies [44, 45]. Even though we did not find evidence that the heterogeneity in the relationship between IADE and CMBs is driven by differences in hypertension in the meta-regression analysis, previous studies have emphasised the strong association between IADE and the presence of CMBs after adjusting for age, sex, and hypertension [13]. Further studies are required to establish the causative relation between CMBs in patients with IADE, especially after correction for the traditional vascular risk factors.

In our systematic review, white matter hyperintensities were observed in 22.8% of ischaemic stroke patients with IADE, over double the risk of WMHs compared to individuals without IADE. A cross-sectional study by Fierini et al. in a cerebrovascular outpatient service confirmed the high prevalence of moderate to severe WMHs in patients with IADE [33]. Subsequent studies have also suggested a relationship between BA dolichoectasia and CSVD [30, 32, 33, 46]. BA diameter significantly correlated with the presence of WMHs, and BA dolichoectasia was approximately three times associated with the severity of WMHs [32]. Although a study of 469 Chinese AIS patients failed to establish the association between IADE and intracranial atherosclerosis (ICAS), they found that IADE had been related to older age, hypertension, multilacunes, and WMHs [30]. An autopsy-based study of 381 patients with stroke emphasised the association of CSVD and its consequence because IADE-positive patients were more significantly to develop SVD than IADE-negative patients. Notably, plaque formation in the affected arteries of IADE patients was elevated considerably, and IADE-positive patients exhibited no evidence of CAA [28]. These findings might be hypothesised that CVSD and IADE in AIS individuals could have common underlying pathophysiologic processes.

Brain atrophy, defined as cortical or subcortical brain volume reduction that is not associated with significant traumatic brain injury or infarction, is recognised as one of the neuroimaging features of CSVD on brain MRI [47–49]. We did not find studies reporting any association between IADE and brain atrophy. However, our results did show an association of IADE with lacunes, which have in turn been associated with regional cortical and subcortical grey matter volume loss in individuals with vascular mild cognitive impairment [50]. Additional studies are required to establish more directly whether IADE is associated with brain atrophy. In addition, it is important to differentiate true lacunar infarcts related to in situ arteriolosclerosis from branch atheromatous disease (BAD), i.e., occlusion of the perforator orifice due to junctional plaque, because the underlying vascular pathology might be different and BAD-related strokes are associated with early neurological deterioration (END) in acute stroke due to mall vessel occlusion [51, 52].

Most previous studies demonstrated the prevalence of IADE or VBD in acute ischaemic stroke populations, while data in intracerebral haemorrhage (ICH)—an important and clinically severe manifestation of CSVD—are limited. In 2012, a study of 481 acute stroke patients showed the prevalence of VBD in patients with ICH was approximately double that seen in patients with brain infarcts (12.1% vs. 6.4%) [53], although the prevalence of VBD in that study was lower than in other studies despite using similar criteria to diagnose VBD [4, 54]. Therefore, additional studies are needed to investigate IADE in patients with ICH. In addition, the utility of VBD as a predictor of stroke outcomes or mortality requires additional investigation. The involvement of the basilar artery was an independent risk factor for transient or fixed posterior circulation dysfunction or neurological morbidity, whereas the mortality in affected patients appears to be anticipated more by traditional vascular risk factors than by VBD characteristics [55]. Increasing evidence is expanding the range of MRI features of lesions related to CSVD, including recent small subcortical infarcts (RSSI), cortical cerebral microinfarcts, and cortical superficial siderosis (cSS); future research concerning associations between these SVD markers and IADE in both ischaemic stroke and ICH may help to better understand the mechanisms underlying the associations we have reported.

In the present study, we comprehensively examined all published reports of associations of IADE with a range of neuroimaging markers of CSVD. However, we acknowledge some limitations, including the small number of included studies that limit our ability to identify publication bias and sources of heterogeneity in meta-regression [56]. Second, the overall quality of the included studies is only fair, and the certainty of evidence for the outcomes is relatively broad, ranging from very low to moderate. Five of seven publications have an NOS score of less than seven. Finally, the effect of gender differences might restrict the applicability of the present outcomes. While several studies found the potential risk of male gender for IADE development in AIS patients, our meta-regression analysis on CMBs outcomes showed a negative relationship between the proportions of male participants and CMBs risk.

## Conclusions

In conclusion, IADE, especially affecting the vertebrobasilar circulation, is consistently associated with CSVD markers, including lacunes, CMBs, and WMHs in patients with ischaemic stroke; this relationship appears to be independent of shared traditional vascular risk factors, suggesting possible shared pathophysiological mechanisms. Further studies, especially in ICH cohorts with coverage of all SVD markers, are needed to further clarify the relationships between CSVD and IADE in patients with stroke.

### Supplementary Information

Below is the link to the electronic supplementary material.

Supplementary file1 (DOCX 211 KB)

## Data Availability

All the datasets generated during the study are available upon reasonable request from the corresponding author.
