# Supplementary material for: Relationships between intracranial arterial dolichoectasia and small vessel disease in patients with ischaemic stroke: a systematic review and meta-analysis
Source: J Neurol. 2023 Nov 29;271(2):772–81. doi: 10.1007/s00415-023-12094-2 (PMC10827828; doi:10.1007/s00415-023-12094-2)
Supplement: Supplementary file 1 — Supplementary file1 (DOCX 211 KB) [file 415_2023_12094_MOESM1_ESM.docx]

**Supplementary information**

**Table S1** Search strategies (March 23, 2023)

| **Database** | **Records** | **Search terms** |
| --- | --- | --- |
| PubMed | 55 | ("dolichoectasia"[MeSH Terms] OR "dolichoectasis"[All Fields] OR "dolichoectatic"[All Fields]) AND ("stroke"[MeSH Terms] OR "stroke"[All Fields] OR "strokes"[All Fields] OR "stroke s"[All Fields] OR "small vessel disease" [MeSH Terms] OR "small vessel diseases"[All Fields]) |
| Embase | 63 | (('dolichoectasia'/exp OR 'dolichoectasia') AND ('small vessel disease'/exp OR 'small vessel disease') AND ('brain haemorrhage'/exp OR 'brain haemorrhage') OR 'brain hemorrhage'/exp OR 'brain hemorrhage') AND dolichoectasia:ti |
| Scopus | 39 | TITLE-ABS-KEY (( dolichoectasia ) OR ( dolichoectasis ) OR ( dolichoectatic )) AND (( small AND vessel AND disease ) OR ( small AND vessel AND diseases ) OR ( cerebral AND small AND vessel AND disease ) OR ( intracerebral AND hemorrhage OR haemorrhage )) |

**Table S2** Quality assessment of enrolled studies according to the Newcastle‐Ottawa Quality Assessment Scale (NOS)

| **Study** | **Selection (0-4)** | | | | **Comparability (0-2)** | | **Outcome (0-3)** | | | **Overall (0-9)** |
| --- | --- | --- | --- | --- | --- | --- | --- | --- | --- | --- |
|  | Is the case definition adequate? | Representativeness of the cases | Selection of Controls | Definition of Controls | Study controls for age | Study controls for gender | Ascertainment of exposure | Same method of ascertainment for cases and controls | Non-Response rate |  |
| Pico et al. 2003 | ★ | ★ | ☆ | ★ | ★ | ★ | ☆ | ★ | ★ | **7** |
| Pico et al. 2005 | ★ | ★ | ☆ | ★ | ★ | ★ | ☆ | ★ | ★ | **7** |
| Pico et al. 2007 | ★ | ★ | ☆ | ★ | ★ | ★ | ☆ | ★ | ☆ | **6** |
| Park et al. 2013 | ★ | ★ | ☆ | ☆ | ★ | ☆ | ★ | ★ | ☆ | **5** |
| Thijs et al. 2017 | ★ | ★ | ☆ | ★ | ★ | ★ | ☆ | ★ | ☆ | **6** |
| Yin et al. 2021 | ★ | ★ | ☆ | ☆ | ★ | ★ | ☆ | ★ | ☆ | **5** |
| Osama et al. 2022 | ★ | ★ | ☆ | ★ | ★ | ★ | ☆ | ★ | ☆ | **6** |

**Table S3** Results from meta-regression analyses to explore heterogeneity induced by the relationship between continuous moderators and cerebral microbleeds.

| **Moderators** | **Coefficient** | **SE** | ***z*** | ***P* value** | **95% CI** | **Residual heterogeneity** | | |
| --- | --- | --- | --- | --- | --- | --- | --- | --- |
|  |  |  |  |  |  | **τ^2^** | ***I*^2^** | ***P* value** |
| Percentages of CMBs in the IADE group | -0.01 | 0.008 | -1.16 | 0.25 | -0.026 to 0.007 | 0.14 | 79.77 | 0.25 |
| NOS | -0.36 | 0.69 | -0.52 | 0.60 | -1.70 to 0.99 | 0.27 | 90.90 | 0.60 |
| Percentages of male participants | -0.05 | 0.03 | -2.01 | 0.045 | -0.11 to -0.00 | 0.05 | 57.43 | 0.045 |
| Percentages of hypertension among participants | 0.02 | 0.03 | 0.55 | 0.58 | -0.04 to 0.07 | 0.27 | 90.56 | 0.58 |
| The mean age of the participants | -0.02 | 0.03 | -0.66 | 0.51 | -0.09 to 0.04 | 0.24 | 88.90 | 0.51 |

**Abbreviations:** CI, confidence interval; CMB, cerebral microbleed; IADE, intracranial arterial dolichoectasia; NOS, the Newcastle-Ottawa Quality Assessment Scale; SE, standard error.

**Table S4** Certainty of evidence for the outcomes

| **Outcome** | **Initial quality of evidence** | **Risk of bias** | **Inconsistency** | **Indirectness** | **Imprecision** | **Publication bias** | **Large effect** | **Dose response** | **Effect of all plausible residual confounding** | **Overall quality level** |
| --- | --- | --- | --- | --- | --- | --- | --- | --- | --- | --- |
| Lacunes | Low (3 observational studies) | No change (6 stars) | No change (*I^2^* = 0.00%) | No change (direct evidence) | No change (significant difference) | No change (*P* = 0.43) | No change (no large RR, 1.67) | No change | No change | Low (⊕⊕○○) |
| CMB | Low (3 observational studies) | No change (6 stars) | -2 (*I^2^* = 84.95%) | No change (direct evidence) | No change (significant difference) | -2 (significant bias, *P* < 0.001) | +1 (large RR, 2.56) | No change | No change | Very low (⊕○○○) |
| WMH | Low (3 observational studies) | No change (6 stars) | No change (*I^2^* = 0.00%) | No change (direct evidence) | No change (significant difference) | No change (*P* = 0.69) | +1 (large RR, 2.17) | No change | No change | Moderate (⊕⊕⊕○) |

**Abbreviations:** CBM, cerebral microbleed; RR, risk ratio; WMH, white matter hyperintensities.

**Fig. S1** Funnel plots for assessing the publication bias of outcomes A) lacunes B) cerebral microbleed C) white matter hyperintensities. CI, confidence interval.

| **A)** 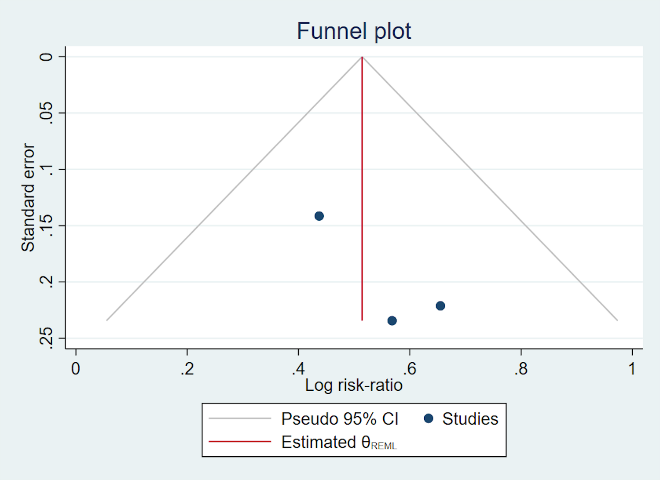 | **B)** 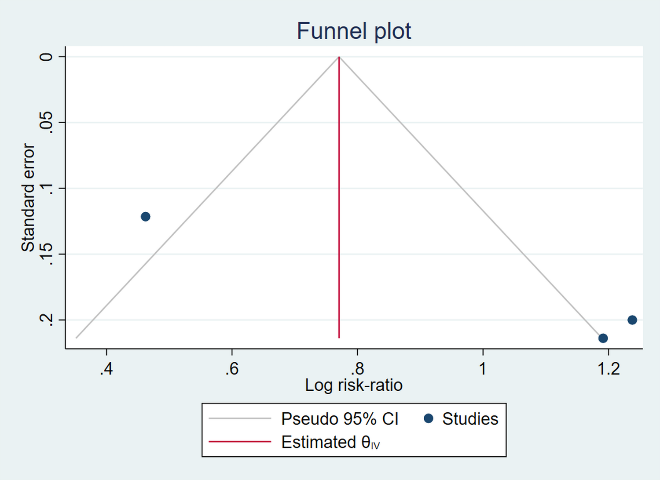 |
| --- | --- |
| **C)** 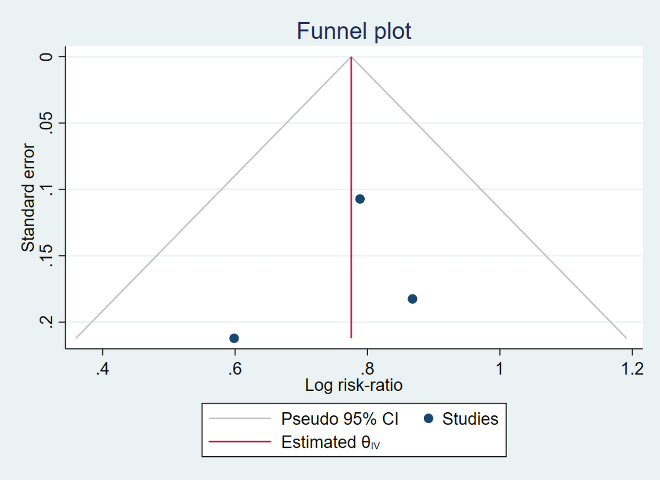 |  |
